# Associations between axial length and optical coherence tomography angiography biomarkers

**DOI:** 10.1186/s40942-025-00763-5

**Published:** 2025-12-29

**Authors:** Jessica Wing Ka Lau, Shing Chuen Chow, Ming Ming Zhu, Pun Yuet Lam, Kendrick Co Shih, Nicholas Siu Kay Fung

**Affiliations:** 1 The Li Ka Shing Faculty of Medicine, The University of Hong Kong, Hong Kong, China; 2 United Christian Hospital, Hong Kong, China; 3 Department of Ophthalmology, Hong Kong Sanatorium & Hospital, 4/F, Li Shu Fan Block, 2 Village Road, Hong Kong, Hong Kong

**Keywords:** Myopia, Optical coherence tomography angiography, Axial length

## Abstract

**Purpose:**

To determine the associations between axial length (AL) and optical coherence tomography angiography (OCTA)-based eye-related parameters.

**Methods:**

A prospective cross-sectional study of the right eye of 6953 individuals between the ages 50 and 97, with a range of AL from 19.1 to 31.7 mm was performed. Central subfield thickness (CST), foveal avascular zone (FAZ) and mean vessel density (mVD) were measured by OCTA. Associations between OCTA-based parameters, eye-related and systemic-related parameters were assessed using one-way ANCOVA, multivariable linear regression analysis and multiple regression analysis.

**Results:**

AL and OCTA-based parameters were negatively correlated with age. Increased AL was associated with increased CST (ß=3.59), decreased FAZ (ß=0.02), and decreased mVD (ß=-0.4) (all *p* = 0.000). Better best corrected visual acuity (BCVA) was associated with larger FAZ (ß=-0.07) and higher mVD (ß=-3.160) (*p* = 0.002, *p* = 0.000). Hypertension and diabetes mellitus correlated with increased mVD (ß=0.5) and decreased mVD (ß=-0.18) respectively (*p* = 0.000, *p* = 0.023).

**Conclusion:**

Increased AL is associated with lower mVD which may be a potential factor between myopia and poor BCVA.

## Introduction

Optical coherence tomography angiography (OCTA) is a novel imaging technology that makes use of dense volumetric scanning to provide depth-resolved images of blood flow in the retina and choroid with levels of detail exceeding traditional angiography [1]. It can create images of all layers of retinal vasculature and create three-dimensional cross-sectional images of the layers of the macular cube. This allows for quantitative analysis of the foveal avascular zone area (FAZ_A) and optic nerve head (ONH) microvasculature [2]. Unlike fluorescein angiography, it is a non-invasive technique that does not require the injection of fluorescent dye, providing a better view of vascular network. Additionally, the superficial and deep peripapillary plexus can be explored easily and separately with OCTA using en face images, whereas fluorescein angiography cannot distinguish between the two plexuses as images are superimposed (two-dimensional images). Furthermore, OCTA has a higher lateral resolution, commonly around 5 to 12 micrometers, because it uses focused light and interferometric detection to capture detailed microvascular structures. This allows OCTA to visualize fine capillary networks and small blood vessels in the retina noninvasively, with high spatial precision in the horizontal plane [3].

In contrast, fluorescein angiography has a lower lateral resolution, generally around 30 to 60 micrometers, as it relies on dye fluorescence visualized with fundus cameras. The resolution is limited by the optics of the camera system and the scattering and spreading of the fluorescent dye, leading to less detailed visualization of smaller vessels compared to OCTA [3–5]. Numerous studies have utilized OCTA to study retinal microvascular changes in various retinopathies and maculopathies and found a significant association between visual acuity and OCTA parameters [6–9].

Myopia is one of the most common refractive errors in the world [10]. Patients with high myopia often have abnormally high axial length and scleral thinning, predisposing them to various diseases including retinal detachment, macula holes, myopic choroidal neovascularization and myopic maculopathy. High myopia is also associated with a higher risk of glaucoma and cataract. However, only several studies have investigated the associations between myopia and OCTA parameters, with relatively small sample size [11, 12]. Despite having pre-existing population-based eye studies [13, 14] on OCT findings of larger patient population, OCTA was not conducted and analyzed in specific. Thus, our study aims at observing the association between axial length and various OCTA parameters in a large population. Furthermore, the association between OCTA findings and other eye related parameters will be discussed.

## Methods

### Study design and patient selection

Data was collected from the University of Hong Kong’s Southern District Community Eye Screening Program, between June 2019 and July 2021. The screening program included participants who live in Hong Kong’s southern district and are 50 years old and above. Each participant was granted one visit to the screening program where they underwent a series of eye examinations for free.

The screening program started with examining autorefraction, BCVA and non-contact tonometry using Nidek Tonoref II, followed by anterior segment OCT using Tomey CASIA 2. Participants would then undergo intra-ocular pressure (IOP) measurement using Icare ic200, followed by corneal keratometry and anterior chamber depth (ACD) measurements using Pentacam AXL and/or Zeiss IOL master 700. Subsequently, the participants were given Mydrin-P for pupil dilation. Thereafter, posterior OCT using Zeiss Cirrus HD-OCT 5000, TOPCON 3D-OCT-1 Maestro 2 and Heidelberg Spectralis. Zeiss Cirrus HD-OCT 5000 scans included macular cube 512 × 128 (1 scan per eye), optic disc cube 200 × 200 (1 scan per eye) and OCT-A macula (1 scan per eye) and disc (1 scan per eye). TOPCON 3D-OCT-1 Maestro 2 obtained 3D Wide (horizontal) 12.0 × 9.0 mm (1 scan per eye). Heidelberg Spectralis scans included macula scan (1 scan per eye) and RNFL (1 scan per eye) scan. The screening program ended with a consultation with an ophthalmologist doing a slit lamp examination.

AL was obtained by the Zeiss IOL master 700 and the Pentacam AXL. ACD was obtained with the Tomey CASIA 2 or the Pentacam AXL. IOP was obtained using the Nidek Tonoref II and the Icare ic200. Central corneal thickness (CCT) was obtained with the Zeiss IOL master 700 or Pentacam AXL.

This study was approved by the HKU/HA/Western Cluster Institutional Review Board (UW18516) and abides by the principles of the *Declaration of Helsinki*. All participants provided informed consent by signing a consent form. All subjects enrolled in the Southern District Community Eye Screening Program during June 2019 to July 2021 were included in the study. The exclusion criteria include participants with retinal drusen, retinal scar, subretinal fluid, retinal hemorrhage, epiretinal membrane, macular hole, atrophy, myopic maculopathy, diabetic retinopathy, retinal break, retinal vein occlusion and those with missing axial length values. The right eye of each participant was selected for statistical analysis to avoid potential bias between both eyes.

### OCTA examination

Trained clinical staff carried out OCTA examinations on all participants using the Carl Zeiss Cirrus 5000 machine (Carl Zeiss Meditec, Inc, Dublin, CA, USA) with Angioplex. First the iris was aligned, then the scan head was advanced until the fundus came into view, ensuring the optic nerve head was clearly visible. OCT alignment was ensured by centering the video image and scan region on the pupil and scan head towards the patient. The working distance was adjusted to optimize the fundus video image. Other settings could be adjusted, such as polarization, Z offset and focus, then the scan was captured. The FastTrac retinal tracking technology helps to minimize motion artifacts by automatically tracking eye movements. After acquisition, en face OCTA images are generated as the software analyzes the scan using the optical microangiography algorithm.

**Fig. 1:**
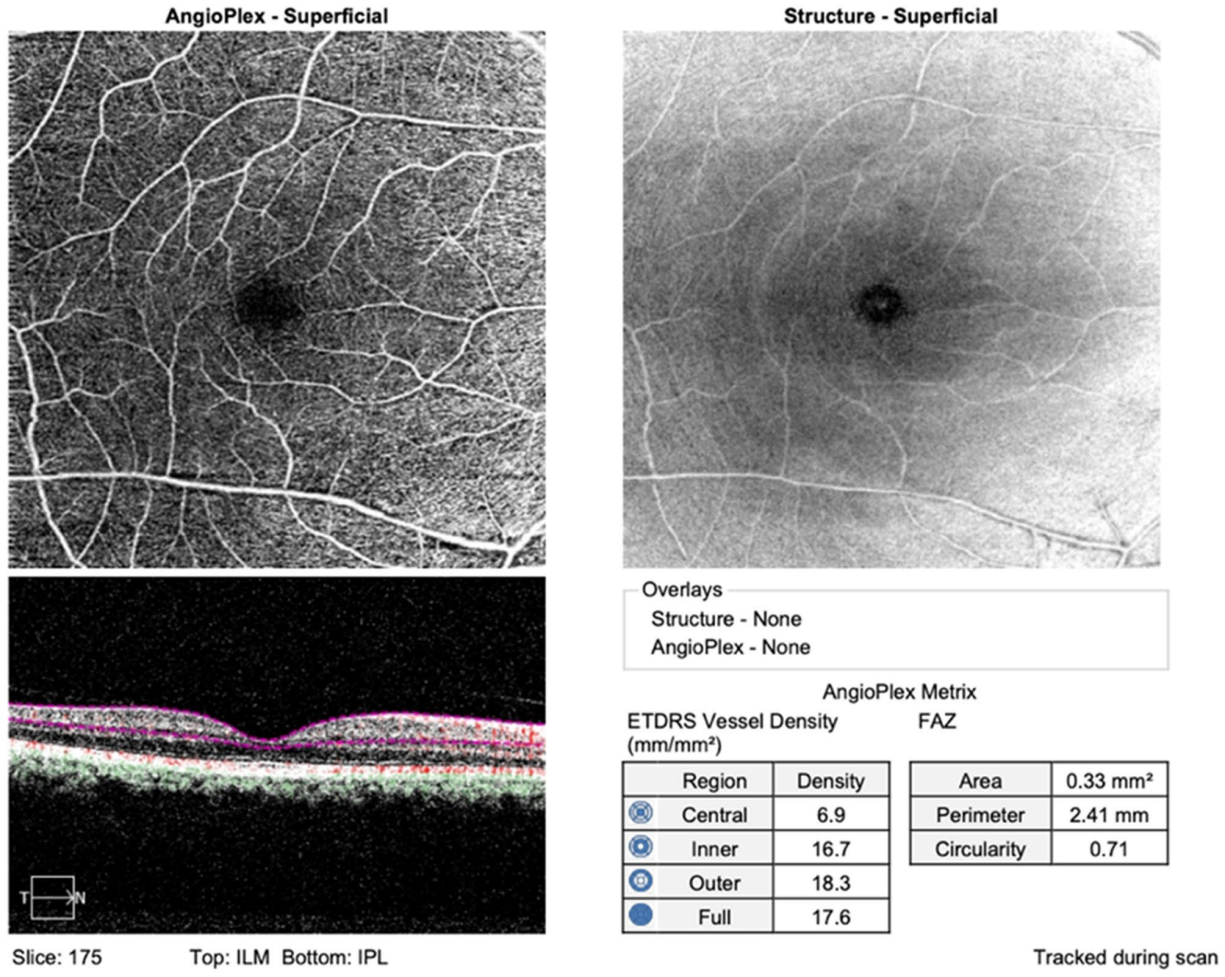
Foveal avascular zone obtained with Carl Zeiss Cirrus 5000 machine. (ILM—Internal limiting membrane; IPL—Inner plexiform layer; FAZ—Foveal avascular zone; ETDRS—Early treatment diabetic retinopathy study)

A real-time eye-tracking system with a 6 × 6 mm scanning model was used to identify the foveal avascular zone area in the superficial capillary plexus (Fig. 1). The superficial capillary plexus is defined as the distance between the inner plexiform layer and the inner limiting membrane [15]. During successive scans, the OCT system acquires multiple B-scans at the same location. By comparing the intensity and phase information between these scans, it can detect changes in signal caused by moving blood cells. The OCTA algorithm analyzes the differences in signal between consecutive B-scans to identify areas of flow and areas of static tissue. These differences create the angiogram. The angiogram data can be visualized in different ways. En face OCTA images provide a two-dimensional view of the retinal or choroidal vasculature at a specific depth. By scanning through different depths, a stack of en face images can be obtained to visualize the vasculature in three dimensions [16]. Ultimately, each OCTA image was assessed by clinical staff to ensure its quality. Only images with a signal strength index of greater than 7 were analyzed.

### Statistical analysis

Statistical analysis was performed using the SPSS software version 26.0. Pearson’s correlation analysis and Spearman’s correlation analysis were performed to analyze the correlation between BCVA, CST_A, FAZ_A, mVD and AL. The difference in means of CST_A, FAZ_A and mVD stratified by AL were estimated by age and adjusted by Bonferroni. The data was analyzed by one-way ANCOVA by setting age as a covariate. Multivariable linear regression analysis was used to explore the independent associations of BCVA. Multiple regression analysis exploring the independent associations of CST_A, FAZ_A and mVD was done. P values of < 0.05 were classified as statistically significant.

## Results

7,717 individuals were recruited for this study; however, 767 individuals were excluded as they fulfilled the exclusion criteria. Thus, 6,950 individuals were analyzed, with their ages ranging from 50 to 97 years old. The mean age of the participants was 63.2 (± 4.8) years. The male to female ratio of the participants was 1: 0.55. The apparent male predominance in this study population was random and not due to selection bias. OCTA images of the right eye of each patient were used for analysis. Demographic, ocular and systemic characteristics are included in Table 1.

**Table 1:** Demographic, ocular and systemic characteristics

Parameters	Number	Mean	Standard Deviation
Age	6950	63.2	4.8
Male vs. female	6950	4485 vs. 2465	
Ocular related parameters			
BCVA (logMAR)	6944	0.1	0.2
AL (mm)	6950	24.2	1.5
CCT (µm)	6948	548.8	34.7
ACD (mm)	6885	3.1	0.5
IOP (mmHg)	3999	14.6	2.9
Systemic related parameters			
Prevalent HT vs. absent	4577	1818 vs. 2759	
Prevalent DM vs. absent	4910	557 vs. 4353	
Prevalence HP vs. absent	4550	1419 vs. 3491	
SBP (mmHg)	6912	135.5	18.8
DBP (mmHg)	6912	82.2	11.2
BMI (kg/m^2^)	6891	24.4	3.7
Height	6896	158.8	8.4
Weight	6891	61.6	11.3
Body fat percentage	6869	31.4	6.1
Visceral fat area	6868	9.2	5.4

BCVA: Best corrected visual acuity; AL: Axial length; CCT: Central corneal thickness; ACD: Anterior chamber depth; IOP: intraocular pressure; HT: hypertension; HP: hyperlipidemia; DM: diabetic mellitus; SBP: systolic blood pressure; DBP: diastolic blood pressure; VFA: Visceral fat area

### Mean value of OCTA parameters and age

6,950 participants were stratified into 6 groups according to their age for analysis (age: 50–55 years, 55–60 years, 60–65 years, 65–70 years, 70–80 years, ≥ 80 years) (Table 2). Shorter axial lengths were observed in older age groups. The mean axial length of the 50–55 age group was 24.53 (± 1.56) mm, when compared to 23.54 (± 1.11) mm in the ≥ 80 age group. CST_A also showed a stepwise decrease from the 50–55 age group (250.68 ± 22.08 μm) to the ≥ 80 age group (248.36 ± 26.9 μm). All three foveal avascular zone measurements (FAZ_A, FAZp, FAZc) were smaller in older patients. The FAZ_A in the youngest group was 0.31 ± 0.15 mm^2^, and it reduced to 0.22 ± 0.15 mm^2^ in the oldest group. All 3 parameters on macular vessel density (mVD_C, mVD_In, mVD_Out) show a decreasing trend with age, which is represented in the decreasing trend of mVD_full as age increases. The full macular vessel density was highest in the 50–55 age group (17.47 ± 1.8 mm/mm^2^), whereas it was the lowest in the ≥ 80 age group (13.62 ± 3.93 mm/mm^2^).

**Table 2:** The mean value of BCVA, AL, CST, FAZ and mVD stratified by age

Parameter	Age						
	50–55	55–60	60–65	65–70	70–80	>=80	All
	N (937–962)	N (1439–1492)	N (1719–1803)	N (1236–1372)	N (906–1082)	N (158–240)	N (6393–6952)
BCVA	0.01 ± 0.1	0.02 ± 0.11	0.05 ± 0.12	0.1 ± 0.17	0.14 ± 0.16	0.28 ± 0.26	0.07 ± 0.15
AL	24.53 ± 1.56	24.46 ± 1.5	24.22 ± 1.44	24.11 ± 1.43	23.91 ± 1.36	23.54 ± 1.11	24.22 ± 1.47
ACD	3.15 ± 0.36	3.11 ± 0.36	3.11 ± 0.44	3.09 ± 0.49	3.11 ± 0.62	3.59 ± 0.96	3.13 ± 0.49
CST_A	250.68 ± 22.08	250.44 ± 21.94	251.07 ± 28.42	249.81 ± 25.33	249.06 ± 39.45	248.36 ± 26.9	250.24 ± 27.78
FAZ_A	0.31 ± 0.15	0.30 ± 0.14	0.28 ± 0.14	0.26 ± 0.13	0.24 ± 0.14	0.22 ± 0.15	0.28 ± 0.14
mVD_C	7.99 ± 3.13	7.49 ± 3.1	7.09 ± 3.1	6.69 ± 3.2	5.86 ± 3.28	5.23 ± 3.64	6.98 ± 3.25
mVD_In	17.18 ± 2.25	16.79 ± 2.44	16.36 ± 2.93	15.85 ± 3.13	14.92 ± 3.7	13.47 ± 4.45	16.16 ± 3.09
mVD_Out	17.91 ± 1.76	17.5 ± 1.96	17.08 ± 2.37	16.52 ± 2.58	15.51 ± 3.03	13.99 ± 3.91	16.85 ± 2.59
mVD_Full	17.47 ± 1.8	17.06 ± 2.01	16.64 ± 2.43	16.1 ± 2.62	15.11 ± 3.1	13.62 ± 3.93	16.42 ± 2.63

BCVA: Best corrected visual acuity; AL: Axial length; ACD: Anterior chamber depth; CST_A: Angiography- Central Subfield Thickness (µm) ILM-RPE; FAZ_A: Foveal avascular zone Area (mm^2^); FAZp: Foveal avascular zone Perimeter (mm); FAZc: Foveal avascular zone Circularity Index; mVD_C: macular Vessel Density (mm/mm^2^) Center; mVD_In: macular Vessel Density (mm/mm^2^) Inner; mVD_Out: macular Vessel Density (mm/mm^2^) Outer; mVD_Full: mean Vessel Density (mm/mm^2^) Full

From the multiple regression analysis in Table 3, the standardized beta coefficient (Beta*) of FAZ_A was − 0.06 per decade increase in age (*p* = 0.00). For mVD_Full, the standardized beta coefficient was − 0.22 per decade increase in age (*p* = 0.00).

**Table 3:** Multiple regression analysis exploring the independent associations of CST, FAZ_A and mVD

Independent Parameters	Dependent Parameters								
	CST_A			FAZ_A			mVD_Full		
	Beta*	Beta (95%CI)	*P*	Beta*	Beta (95% CI)	*P*	Beta*	Beta (95%CI)	*P*
Constant		159.7 (127.5, 191.89)	0.000		0.93 (0.73, 1.13)	0.000		30.63 (27.42, 33.85)	0.000
Per decade in age	0.00	-0.14 (-1.31, 1.03)	0.815	-0.17	-0.03 (-0.04, -0.02)	0.000	-0.37	-1.21 (-1.33, -1.09)	0.000
Male vs. female	0.18	9.39 (5.82, 12.96)	0.000	-0.04	-0.01 (-0.03, 0.01)	0.264	0.00	0 (-0.36, 0.36)	1.000
Eye related parameters									
Per 1 μm in AL	0.17	2.89 (2.31, 3.47)	0.000	-0.15	-0.02 (-0.02, -0.01)	0.000	-0.24	-0.43 (-0.49, -0.37)	0.000
Per 10 μm in CCT	0.01	0.21 (-0.51, 0.93)	0.565	-0.02	0 (-0.01, 0)	0.180	0.02	0.04 (-0.03, 0.11)	0.240
Per 1 μm in ACD	0.08	3.84 (2.23, 5.45)	0.000	0.02	0.01 (-0.01, 0.02)	0.327	0.06	0.3 (0.14, 0.46)	0.000
Per 5mmHg in IOP	-0.01	-0.59 (-2.09, 0.92)	0.444	0.00	0 (-0.01, 0.01)	0.942	-0.03	-0.14 (-0.29, 0.01)	0.074
Systemic related parameters									
Per 2 kg/m^2^ in BMI	-0.05	-0.62 (-2.03, 0.79)	0.390	-0.05	0 (-0.01, 0.01)	0.428	0.03	0.04 (-0.1, 0.18)	0.606
Prevalent HT	0.02	0.63 (-0.96, 2.22)	0.440	0.03	0 (-0.01, 0.01)	0.392	0.22	0.65 (0.49, 0.81)	0.000
Prevalent DM	0.04	0.93 (-0.96, 2.82)	0.334	-0.01	0 (-0.01, 0.01)	0.780	-0.08	-0.22 (-0.41, -0.03)	0.020
Prevalent HP	-0.02	-0.53 (-2.65, 1.59)	0.622	0.04	0.01 (-0.01, 0.02)	0.360	0.04	0.11 (-0.1, 0.32)	0.314
Per 10 mmHg in SBP	-0.03	-0.42 (-1.03, 0.18)	0.168	0.03	0 (0, 0.01)	0.179	0.00	-0.01 (-0.07, 0.05)	0.855
Per 10 mmHg in DBP	-0.01	-0.11 (-1.09, 0.86)	0.819	-0.01	0 (-0.01, 0)	0.538	-0.01	-0.02 (-0.12, 0.08)	0.689
Per 10 cm in Height	0.03	0.92 (-0.4, 2.24)	0.170	-0.02	0 (-0.01, 0.01)	0.500	0.04	0.13 (0, 0.27)	0.045
Per 5% in Body fat	0.00	0.08 (-1.09, 1.25)	0.888	0.03	0 (0, 0.01)	0.364	-0.02	-0.04 (-0.16, 0.08)	0.494
VFA	0.04	0.19 (-0.31, 0.7)	0.454	0.01	0 (0, 0)	0.903	-0.02	-0.01 (-0.06, 0.04)	0.764
Model summary	Adjusted R^2^ = 0.11, F = 31.83, *p* = 0.000			Adjusted R^2^ = 0.05, F = 13.06, *p* = 0.000			Adjusted R^2^ = 0.18, F = 54.11, *p* = 0.000		

AL: Axial length; ACD: Anterior chamber depth; CCT: Central corneal thickness; IOP: intraocular pressure; BMI: Body mass index; HT: hypertension; DM: diabetic mellitus; HP: hyperlipidemia; SBP: systolic blood pressure; DBP: diastolic blood pressure; VFA: Visceral fat area

The choice to set the independent parameter as per 1 μm in AL reflects the need for precision and sensitivity in quantifying the relationship between small changes in axial length and OCTA parameters. Using 1 μm increments provides more granular insight into how minimal axial length variations correlate with OCTA metrics, which is crucial for detailed ophthalmic research.

Included eyes were divided into four groups in terms of axial length (group A: AL < 22, group B: 22 ≤ AL < 24, group C:24 ≤ AL < 26, group D AL ≥ 26) (Table 4). A total 187 eyes were included in group A, 3379 eyes in group B, 2526 eyes in group C and 858 eyes in group D.

**Table 4:** The difference in means of CST, FAZ_A and mVD stratified by AL

	Mean ± SD				Test between subjects’ effects*	Pairwise comparisons^&^		
	Group A AL < 22 *N* = 187	Group B 22 ≤ AL < 24 *N* = 3379	Group C 24 ≤ AL < 26 *N* = 2526	Group D AL ≥ 26 *N* = 858		Groups	Mean difference (95%CI)	P value
CST_A	241.6 ± 1.87 *N* = 173	244.78 ± 0.43 *N* = 3309	254.22 ± 0.5 *N* = 2480	260.78 ± 0.86 *N* = 831	Corrected model: F = 100.05; *P* = 0.000 Age: F = 0.59; *P* = 0.80 AL: F = 117.7; *P* = 0.000	A vs. B	-3.15(-8.22, 1.90)	0.60
						A vs. C	-12.60(-17.71, -7.50)	0.000
						A vs. D	-19.16 (-24.60, -13.72)	0.000
						B vs. C	-9.45(-11.18, -7.71)	0.000
						B vs. D	-16.00(-18.55, -13.45)	0.000
						C vs. D	-6.56(-9.16, -3.95)	0.000
FAZ_A	0.30 ± 0.01 *N* = 167	0.29 ± 0.00 *N* = 3112	0.26 ± 0.00 *N* = 2347	0.23 ± 0.01 *N* = 766	Corrected model: F = 72.55; *P* = 0.000 Age: F = 154.42; *P* = 0.000 AL: F = 60.95; *P* = 0.000	A vs. B	0.01(-0.02, 0.04)	0.03
						A vs. C	0.04(0.01, 0.07)	0.002
						A vs. D	0.08(0.05, 0.11)	0.000
						B vs. C	0.03(0.02, 0.04)	0.17
						B vs. D	0.07(0.05, 0.08)	0.003
						C vs. D	0.03(0.02, 0.05)	0.000
mVD_Full	16.91 ± 0.18 *N* = 176	16.74 ± 0.04 *N* = 3312	16.41 ± 0.05 *N* = 2485	15.04 ± 0.09 *N* = 832	Corrected model: F = 303.07; *P* = 0.000 Age: F = 1030.08; *P* = 0.000 AL: F = 108.10; *P* = 0.000	A vs. B	0.17(-0.32, 0.67)	1.00
						A vs. C	0.50(-0.00, 1.00)	0.51
						A vs. D	1.87(1.33, 2.40)	0.000
						B vs. C	0.33(0.16, 0.50)	0.000
						B vs. D	1.70(1.44, 1.95)	0.000
						C vs. D	1.37(1.11, 1.63)	0.000

CST_A: Angiography- Central Subfield Thickness (µm) ILM-RPE; FAZ_A: Foveal avascular zone Area (mm2); mVD_Full: mean Vessel Density (mm/mm2) Full

### Angiography- central subfield thickness

By performing pairwise comparison, significant differences were found between groups A, B, C and D, apart from the comparison between group A and group B, where the difference was insignificant. All the significant results showed that CST_A increases when axial length increases (*P* = 0.000). An increasing trend was also shown in group A and B, CST_A increased from 241.6 ± 1.87 to 244.78 ± 0.43 μm, however, no significance was found in this subgroup comparison (*P* = 0.60). Multiple regression analysis (Table 3) showed that the standardized (regression) coefficients by setting per 1 μm of axial length as an independent parameter and CST _A was 0.17 (*P* = 0.000). One-way ANCOVA was performed to adjust CST_A with age and axial length, the result showed that age does not have a significant effect (*p* = 0.44) while axial length has a significant effect on CST_A (*P* = 0.000).

### Foveal avascular zone area

Apart from comparison between group B and C, pairwise comparison between group A, B, C, D showed significant decrease in FAZ_A when axial length increases (*P* = 0.000). A significant decrease in 0.01mm^2^ was found when comparing group A to group B (*P* = 0.03). A comparison between group C and D shows a significant decrease of 0.01 mm^2^ (*P* = 0.000). An insignificant decreasing trend was found between group B and C (0.29mm^2^ vs. 0.26mm^2^) (*P* = 0.17). By adjusting FAZ_A with age and axial length using one-way ANCOVA, both were found to have significant effects on FAZ_A (*P* = 0.000).

### Mean vessel density full

Pairwise comparison showed a significant decrease in mVD_Full when axial length increases amongst group A, B, C and D (*P* = 0.000), except for comparisons between A and B, and A and C, which showed an insignificant decrease in mVD_Full. One-way ANCOVA indicates that both age and axial length were found to have significant effects on mVD_Full (*P* = 0.000).

### Independent association of BCVA

A significant correlation was found between FAZ_A and BCVA by performing multivariable linear regression analysis. FAZ_A had a significant negative standardized beta coefficient with BCVA, being − 0.05 (*P* = 0.002) when setting FAZ_A as an independent parameter. In addition, mVD_Full also had a significant negative standardized beta coefficient with BCVA, identified as − 0.18 (*P* = 0.000) when mVD_Full is set as an independent parameter. Moreover, per 10 cm increase in height, a significant negative standardized beta coefficient − 0.07 (*P* = 0.002) with BCVA was observed. A significant correlation was also found between age and BCVA. By increasing a decade in age as an independent parameter, a significant positive standardized beta coefficient of 0.34 was found in BCVA (*P* = 0.000). No significant correlation between other systemic related parameters (BMI, hypertension, diabetes mellitus, hyperlipidaemia, systolic blood pressure, diastolic blood pressure, VFA, body fat), nor in other eye related parameters (CST_A, AL, CCT, ACD, IOP) were found in multivariable linear regression analysis (Table 5).

**Table 5:** Multivariable linear regression analysis exploring the independent associations of BCVA

Independent Parameters	Dependent Parameter: BCVA (LogMAR)		
	Beta*	Beta (95%CI)	P
Constant		0.09 (-0.07, 0.25)	0.282
Per decade in age	0.34	0.05 (0.05, 0.06)	0.000
Male vs. female	-0.06	-0.02 (-0.03, 0)	0.07
**Eye related parameters**			
Per 1 μm in AL	-0.01	0 (0, 0)	0.698
Per 10 μm in CCT	-0.02	0 (0, 0)	0.185
Per 1 μm in ACD	0.01	0 (-0.01, 0.01)	0.698
Per 5mmHg in IOP	0.00	0 (-0.01, 0.01)	0.95
CST_A	-0.02	0 (0, 0)	0.373
FAZ_A	-0.05	-0.04 (-0.07, -0.02)	0.002
mVD_Full	-0.18	-0.01 (-0.01, -0.01)	0.000
**Systemic related parameters**			
Per 2 kg/m^2^ in BMI	0.09	0.01 (0, 0.01)	0.085
Prevalent HT	-0.03	0 (-0.01, 0)	0.297
Prevalent DM	0.01	0 (-0.01, 0.01)	0.789
Prevalent HP	0.00	0 (-0.01, 0.01)	0.93
Per 10 mmHg in SBP	0.02	0 (0, 0)	0.471
Per 10 mmHg in DBP	-0.01	0 (-0.01, 0)	0.579
Per 10 cm in Height	-0.07	-0.01 (-0.02, 0)	0.002
Per 5% in Body fat	-0.02	0 (-0.01, 0)	0.594
VFA	-0.03	0 (0, 0)	0.542
Model summary	Adjusted R^2^ = 0.22, F = 53.41, *p* = 0.000		

AL: Axial length; CCT: Central corneal thickness; ACD: Anterior chamber depth; IOP: intraocular pressure; CST_A: Angiography- Central Subfield Thickness (µm) ILM-RPE; FAZ_A: Foveal avascular zone Area (mm2); mVD_Full: mean Vessel Density (mm/mm2) Full; BMI: Body mass index (kg/m^2^); HT: hypertension; DM: diabetic mellitus; HP: hyperlipidemia; SBP: systolic blood pressure (mmHg); DBP: diastolic blood pressure (mmHg); VFA: Visceral fat area; Beta*: standardized beta coefficient

### Independent association of CST_A, FAZ_A and mVD Full

#### Angiography-central subfield thickness

A significant correlation was found between anterior chamber depth (ACD) and CST_A by performing multiple regression analysis. By increasing 1 μm in ACD as an independent parameter, a significant positive standardized beta coefficient of 0.08 was found in CST_A (*P* = 0.000). No significant correlation between other systemic related parameters (BMI, hypertension, diabetes mellitus, hyperlipidaemia, systolic blood pressure, diastolic blood pressure, height, VFA, body fat) and CST_A were found in multiple regression analysis.

#### Foveal avascular zone area

AL had a significant negative standardized beta coefficient with FAZ_A. The significant negative standardized beta coefficient by setting per 1 μm in axial length as an independent parameter is − 0.15 (*P* = 0.000).

#### Mean vessel density full

AL and DM prevalence were found to have a negative correlation with mVD_Full when performing multiple regression analysis. The standardized beta coefficient between per 1 μm in AL and mVD_Full was − 0.24 (*P* = 0.000). The standardized beta coefficient between DM prevalence and mVD full was found to be -0.08 (*P* = 0.020). Prevalence of hypertension and mVD_Full was also found to have a significant positive correlation, with the standardized beta coefficient identified as 0.22 (*P* = 0.000).

## Discussion

Our study is the first prospective cross-sectional study with a vast sample size, providing a normative database on OCTA parameters of Hong Kong citizens who are 50 and above.

This study identified that with an increasing AL, FAZ_A and mVD decreases, while CST increases. Wang et al. found a negative correlation between AL and FAZ_A [17]. One possible explanation is myopia is associated with alterations in retinal blood flow and oxygen metabolism and can lead to vascular remodeling and contraction of the FAZ_A [18, 19]. However, there are other studies that indicate otherwise [20–23]. Piao et al. established that FAZ_A increases when AL increases. They hypothesize that the thinning of the macula that occurs in myopic eyes reduces oxygen consumption thereby reducing retinal blood flow, increasing FAZ_A [20]. The discrepancy may arise from their small sample size of 106 eyes compared to the 6950 eyes in this study. Additionally, their age range focused on the younger population, being 18–34 years compared to 50–97 years in this study. He et al., also has similar results, but the sample size was 760 participants, and the study only included university students in China, between 18 and 25 years old [22]. Similar results were demonstrated in two other studies [21, 23], with a small sample size and focused on younger population (97 subjects and 145 subjects respectively). Li et al. suggested no significant difference in FAZ_A between myopic and normal eyes with a relatively small sample size (40 subjects) [24].

In this present study, mVD is negatively correlated with AL, but the relationship is insignificant in the comparison between groups A and B, and A and C (Table 3). This could be because there are much less participants in group A compared to groups B, C and D. The negative correlation between mVD and AL is consistent with previous studies that have reached similar conclusions [17, 25–27]. Živković et al. identified a significant decrease in mVD in myopic eyes compared to the control group. They theorize that the thinning of the retina from mechanical stretching causes retinal atrophy, reducing its oxygen consumption [28]. The need for less oxygen translates to less vessels required which ultimately reduces vessel density. Another proposed mechanism is that a thinner retina allows for easier diffusion of oxygen from the choroid, causing a secondary loss of vessels [17, 28]. Vascular endothelial growth factor may also play a role [29]. However, Yang et al. did not identify any decrease in macular vessel density in their high myopia group. The disagreement may arise from their small age span and younger cohort (18–32 years old) being assessed, as well as their smaller sample size of 268 eyes [30]. Central subfield thickness primarily reflects the thickness of the central macular region and is commonly used to evaluate retinal conditions such as diabetic macular edema and age-related macular degeneration. Although CST is not a direct measurement of myopia itself, there may be some associations between CST and changes in axial length. The underlying reason could be due to structural changes in myopia such as the thinning of the macular tissue and the development of complications such as choroidal neovascularization or macular atrophy, can potentially result in alterations in CST. In some cases of myopia, there may be associated vitreoretinal traction, giving rise to conditions such as myopic traction maculopathy, which can cause retinal thickening or distortion in the central macular region, affecting CST. Myopia can also be associated with other retinal conditions, namely retinal detachment or epiretinal membrane, which can also affect CST.

This study identified that higher mVD and larger FAZ_A is associated with better BCVA. Multiple studies had found that mVD was negatively correlated with LogMAR BCVA [31–34], supporting the findings of this study. Although, their study population included patients who suffered from various underlying eye conditions including acute primary angle-closure glaucoma, diabetic retinopathy and thyroid-associated ophthalmopathy. The proposed mechanism suggests that a reduction in vessel density may result in ischemia, insufficient oxygen and nutrient supply to the retina and macula, causing photoreceptor and macular cell dysfunction or even cell death, worsening visual acuity [31, 32]. However, a study conducted by Hajdu et al. found no significant correlation between mVD and BCVA [35]. Possible explanations for this discrepancy include the difference in study population and small sample size. Hajdu et al. assessed eyes with either diabetic retinopathy or retinal vein occlusion and had a sample size of 44 eyes [35]. Kim et al. conducted a study on patients with epiretinal membrane, discovering that FAZ_A had a significant negative correlation with BCVA, in agreement with this study. A contracted FAZ resulting in increased mechanical stress on the outer retina could be a cause of the reduced visual acuity [36]. Despite this, Zhang et al. and Dong et al. found no significant correlation between FAZ_A and BCVA [32, 37]. This could be explained by the difference in study population, with Zhang et al. studying eyes with thyroid associated ophthalmopathy [32]. For Dong et al., all the participants were self-reported Caucasians and Afro-Americans. Most of our participants were Asians [37]. Additionally, both had a much smaller sample size of 70 patients and 759 patients respectively. A younger population was also studied in Zhang et al., recruiting 23- to 50-year-olds [32].

The exceptionally large sample size of 6950 eyes in our study allows greater statistical power to detect significant effects and reduces the risk of false positives or negatives. It enhances the generalizability or external validity of the study findings and leads to narrower confidence intervals and more precise estimates of the measures of effect or association. Another strength would be the wide age range (50–97 years). This allows for enhanced generalizability and enables the identification of any developmental trajectories, age-related effects, or associations.

Moreover, the homogeneity of the study population – mainly Chinese participants, may reduce confounding variability related to ethnic differences in ocular anatomy and vascular characteristics, which are known to influence OCTA metrics. This can improve the internal validity and allow for more precise characterization of associations specific to Asian eyes, which is valuable given the high prevalence of myopia in Asian populations. However, the predominance of Asian participants also limits the generalizability of the findings to other ethnic groups, as retinal microvascular features and axial length distributions differ among ethnicities [38]. Therefore, while the findings are highly relevant for the local population, caution should be exercised when extrapolating results to more ethnically diverse or non-Asian populations, emphasizing the need for future multiethnic studies to validate and extend these associations.

For limitations, we recognize that OCTA requires great cooperation and patience from participants which may affect the accuracy and interpretation of the results. Additionally, the quality of OCTA images can vary among individuals. Variability in image quality can affect the quantification and comparability of the OCTA data. Since this is a cross-sectional study, it is not possible to see how FAZ_A, mVD and CST changes after. Axial length variation has been associated with image magnification errors in OCTA; hence, caution must be taken when interpreting and comparing ocular parameters derived from OCTA without image size correction. Another limitation to our study was our lack of data on choroidal thickness. Choroidal thickness is widely reported to be inversely correlated with AL and age in numerous studies. Longer AL corresponds to thinner choroidal measurements, as the elongation of the eye structurally thins the choroid across macular regions, particularly in the central fovea (correlation coefficients ranging approximately from − 16 to -27 μm per mm increase in AL). Age-related thinning of the choroid is also evident, especially after age 50, though the association is generally weaker than that with AL, with yearly decreases in choroidal thickness reported from about − 1.3 to -5.5 μm depending on age group and macular region. This inverse relationship reflects degeneration or reduced vascular support in the choroid with aging and ocular elongation. These associations are relevant to the research on the associations between AL and OCTA parameters because choroidal thickness influences the microvascular environment captured by OCTA. Since axial elongation leads to thinner choroid, it is expected to affect OCTA-derived parameters of choroidal and retinal vascular perfusion. Understanding how AL correlates with choroidal thickness across ages helps interpret OCTA vascular density and flow metrics, enabling better characterization of microvascular changes in myopic eyes or aging populations. Thus, investigating the associations between AL and OCTA necessitates accounting for choroidal thickness as an anatomically and physiologically important mediator in the chorioretinal microvasculature [39].

Another constraint in this study was not excluding patients who have undergone refractive surgery. This could introduce several confounding factors that affect the validity of our findings. Refractive surgeries alter the corneal curvature and thickness without directly changing the axial length. However, these surgeries may affect measurements related to ocular biometry and optical properties critical to OCTA imaging. For example, corneal reshaping can influence the quality and accuracy of OCTA images due to changes in light refraction and signal strength. Furthermore, refractive surgery patients may have altered retinal and choroidal microvasculature due to surgical trauma or changes in ocular physiology post-surgery, which may confound the relationship between AL and OCTA vascular parameters. Including such patients may dilute or obscure true associations by introducing variability unrelated to AL itself [40].

In conclusion, our study found that increasing axial length is associated with decreased mVD and FAZ_A, while CST increases. Higher mVD and larger FAZ_A is associated with better BCVA. This highlights the possible pathophysiology of why visual acuity is reduced in myopia. Clinically, longitudinal OCTA imaging can help monitor and assess the progression of myopia-related complications. Early detection of changes through OCTA can facilitate timely intervention and management. Ultimately, this study provides interesting results on a large population aged 50 and above from Hong Kong, providing normative data in this population.

## Data Availability

Data available by request to corresponding author up to 3 years after publications.

## Abbreviations

BCVA: Best corrected visual acuity
AL: Axial length
CCT: Central corneal thickness
ACD: Anterior chamber depth
IOP: Intraocular pressure
HT: Hypertension
HP: Hyperlipidemia
DM: Diabetic mellitus
SBP: Systolic blood pressure
DBP: Diastolic blood pressure
VFA: Visceral fat area
CST_A: Angiography- Central Subfield Thickness (µm) ILM-RPE
FAZ_A: Foveal avascular zone Area (mm^2^)
FAZp: Foveal avascular zone Perimeter (mm)
FAZc: Foveal avascular zone Circularity Index
mVD_C: Macular Vessel Density (mm/mm^2^) Center
mVD_In: Macular Vessel Density (mm/mm^2^) Inner
mVD_Out: Macula Vessel Density (mm/mm^2^) Outer
mVD_Full: Mean Vessel Density (mm/mm^2^) Full
BMI: Body mass index
HT: hypertension
DM: Diabetic mellitus
HP: Hyperlipidemia
SBP: Systolic blood pressure
DBP: Diastolic blood pressure
VFA: Visceral fat area
